# Quality changes in Chinese high-quality indica rice under different storage temperatures with varying initial moisture contents

**DOI:** 10.3389/fnut.2024.1334809

**Published:** 2024-03-11

**Authors:** Dongyi Zhu, Tingting Wang, Xiuying Liu, Jie Bi, Wei Zhang, Xuefeng Zeng, Pingping Wang, Zaixi Shu

**Affiliations:** ^1^College of Food Science and Engineering, Wuhan Polytechnic University, Wuhan, China; ^2^Key Laboratory for Deep Processing of Major Grain and Oil (Wuhan Polytechnic University), Ministry of Education, Wuhan, China; ^3^School of Liquor and Food Engineering, Guizhou University, Guiyang, China

**Keywords:** rice, storage, temperature, quality, moisture content

## Abstract

The planting area of high-quality indica rice varieties has been growing rapidly in China. However, the storage characteristics of these varieties remains unclear. In this research, different moisture contents (13.5, 14.5, and 15.5%) of high-quality rice (variety Xiadao No.1) were stored at different temperatures (15, 20, 25, and 30°C) for 360 d, and then evaluated for lipid metabolism, redox enzyme activities, fatty acid composition, and sensory attributes. With the prolongation of storage, rice displayed an upward trend in fatty acid value, malondialdehyde content, and cooked rice hardness and a downward trend in contents of total fat and non-starch lipid, peroxidase and catalase activities, and sensory score of cooked rice. The change trends of these quality parameters were aggravated by elevating storage temperature and moisture content. Linoleic acid content of rice generally decreased with prolonged storage. After 300 d of storage, rice with initial moisture content of 13.5% at 30°C showed a fatty acid value of higher than 30 mg KOH/100 g, while rice of other two initial moisture contents reached similar level at 25°C. After the whole storage period, only rice with initial moisture contents of 13.5 and 14.5% stored at 15°C had a sensory score of higher than 60. These results suggested that the aging process of high-quality rice can be inhibited by decreasing the storage temperature and initial moisture content. These results can provide reference for grain storage enterprises to select proper storage condition to store high-quality rice.

## 1 Introduction

Rice is considered one of the three primary crops globally, alongside wheat and corn. The global agricultural land covers approximately 160 million hectares, serving as a source of sustenance for approximately 3.5 billion individuals worldwide (1). Asia is responsible for 90% of global rice production and consumption, with China holding the position of the largest producer and consumer of rice worldwide (2). The Asian cultivated rice can be divided into two subspecies of rice, indica and japonica. Indica rice is more suitable for growth in tropical and subtropical regions, while japonica rice is mainly distributed in temperate and cooler regions (3, 4).

In recent years, there has been a growing focus on the issue of food security (5, 6). Natural disasters are an important factor affecting food production. Natural disasters such as earthquakes, floods and typhoons can cause great damage to cereal crop production (7, 8). The volatility of the global geopolitical landscape can also exert influence on food availability, since instances of political instability, armed conflict, and other related events can potentially result in regional food supply tensions (9). Grain reserves have emerged as a significant strategy in numerous nations to safeguard food security (10, 11). The grain reserve system in China has a significant historical background, dating back over two millennia. In China, rice is the predominant type of reserve grain, with storage duration of up to 3 years.

The physical and chemical properties of rice will undergo a series of changes with the extension of storage time (12, 13). This process of change is known as aging (14). Changes in lipids are also considered to be the most crucial factor leading to rice aging. There are two key processes involved in lipid metabolism. One is that lipids are hydrolyzed by lipase to produce free fatty acids, and the other is that lipoxygenase oxidizes lipids (including free fatty acids) to hydroperoxides (15). The enzyme activity of rice can be affected by the storage process. Zhao et al. (16) reported that the activities of lipase and catalase of rice were inhibited after storage. Storage will lead to the decline in the eating quality of rice. Rice varieties, storage temperature, moisture content and many other factors can affect the aging process of rice during storage, and temperature is generally considered to be the key factor (17, 18).

In recent years, rice breeding technology has developed rapidly and the shift in attention from yield-centric approaches to a more comprehensive consideration of taste has led to the emergence of high-quality cultivars with favorable taste profiles (19). In China, indica rice varieties with the following quality characteristics is called high-quality variety: head rice rate ≥ 44%, chalkiness ≤8%, imperfect grain content ≤5%, amylose content 14.0% ~ 24%, and sensory score ≥ 70. At present, high-quality indica rice has gradually replaced traditional variety as the mainstream planting variety. However, storage enterprises have found that the quality of such rice is easy to deteriorate and cannot meet the requirements of long-term storage. Although rice grains contain a much smaller proportion of lipids than starch, rice lipids were supposed to make a significant contribution to processing and nutritional properties (20). Until now, there have been rare systematic studies reported on lipid metabolism and quality of high-quality rice during storage. The purpose of this study was to investigate the effect of initial moisture content and storage temperature on the lipid oxidation and quality attributes of high-quality rice cultivar in China. In this study, different initial moisture contents (13.5, 14.5, and 15.5%) of high-quality rice were stored at different temperature conditions (15, 20, 25, 30°C) for 360 days and the stored rice samples were evaluated for lipid oxidation-related indicators, cooking, and sensory attributes. This research can provide theoretical and technical guidance for grain storage enterprise to better preserve Chinese high-quality rice.

## 2 Materials and methods

### 2.1 Reagents and samples

Potassium hydroxide, phenolphthalein, EDTA disodium salt dihydrate, thiobarbituric acid, boric acid, linoleic acid, ethyl ether, hydrogen peroxide solution (w/w, 30%), and sodium tetraborate were purchased from China National Pharmaceutical Group Chemical Reagent Co., Ltd. (Shanghai, China). Creosote was purchased from Tianjin Comio Chemical Reagents Co., Ltd. (Tianjin, China). Potassium hydrogen phthalate was purchased from Shanghai Shanpu Chemical Industry Co., Ltd. (Shanghai, China).

The samples used in this study were Chinese indica rice (Xiadao No. 1) harvested on October 9th, 2021, from Xiantao City, Hubei Province, China.

### 2.2 Storage conditions of the rice grains

The rice grains were divided into three batches, with each batch adjusted to the initial moisture content of 13.5, 14.5, and 15.5%, respectively. Each batch containing 3 kg of rice grains was then packed in polyethylene pouches. The samples were stored under various temperatures (15, 20, 25, and 30°C) for a duration of 360 days.

### 2.3 Determinations of physicochemical indicators of the rice samples

During storage of the rice grains samples, indicators related to rice quality were regularly measured. The interval for measurements was 60 days. The fatty acid value (FAV) of rice was determined according to the Chinese standard method of GB/T 20569–2006. In brief, the fatty acid of brown rice flour was extracted with absolute ethanol and then titrated with potassium hydroxide standard solution by using phenolphthalein as indicator.

The malondialdehyde (MDA) content of rice were determined according to the Chinese standard method of GB/T 5009.181–2016. In brief, the brown rice flour was extracted with trichloroacetic acid and then centrifuged. The supernatant was added with thiobarbituric acid for reaction. The reaction solution was measured spectrophotometricaly at 532 nm to determine the MDA content.

The crude fat content in rice was analyzed by the Soxhlet extraction method following the Chinese standard method of GB/T 5009.6–2016. Absolute ethyl ether was used as extraction solvent.

The non-starch lipid (NSL) content was determined according to method of Zhang et al. (21) with some modification. Briefly, 4 g of brown rice flour was added to a mixture solution of 50 mL chloroform and methanol (v/v, 2:1). The mixture was vigorously shaken and allowed to react for 4 h. The supernatant was collected and subjected to evaporation, followed by drying and weighing to determine the content of NSL.

### 2.4 Detection of enzyme activities

#### 2.4.1 Lipoxygenase

The substrate solution was prepared by mixing 0.4 mL of linoleic acid with 0.5 mL of 10% NaOH, and then making up the volume to 100 mL. Accordingly, 20 mL of the above solution was mixed with 0.1 mL of Tween 60, and then supplemented to a total volume of 100 mL using 0.2 mol/L borate buffer (pH 9.0) to create a stock solution. Before using, the stock solution should be diluted 40-fold.

To extract the lipoxygenase (LOX) in samples, 1 g of the powdered sample was mixed with 25 mL of 0.1 mol/L phosphate buffer (pH 7.5), and shaken at 4°C for 30 min. Afterward, the mixture was centrifuged at 8,000 rpm for 10 min, and the supernatant was collected as enzyme extraction.

To detect the LOX activity, 2.8 mL of the substrate solution was combined with 0.2 mL of the enzyme extraction. The mixture was thoroughly mixed, and the absorbance was measured at 234 nm using a UV–Visible Spectrophotometer (T6, PUXI, Beijing, China).

#### 2.4.2 Catalase

The catalase (CAT) activity was detected according to the Chinese standard Methods of GB/T 5522–2008. In brief, catalase was extracted from brown rice flour by using Sorensen’s sodium phosphate buffer. The enzyme solution was added to hydrogen peroxide solution for reaction. The reaction was then terminated using 95% sulfuric acid. The remaining hydrogen peroxide of the reaction solution was titrated using potassium permanganate to calculate the CAT activity.

#### 2.4.3 Peroxidase

To extract the peroxidase (POD) in samples, 1 g of powdered sample was mixed with 10 mL of 0.05 mol/L KH_2_PO_4_ solution, and ground into a homogeneous slurry. The slurry was centrifuged at 4000 rpm for 15 min. After centrifugation, 5 mL of the supernatant was collected, and then made up to 10 mL with KH_2_PO_4_ solution as the enzyme extraction.

The substrate solution was prepared by adding 56 μL of guaiacol into 0.1 mol/L phosphate buffer (pH 6.0), and heating it on a magnetic stirring at 40°C for 20 min. After cooling to room temperature, 38 μL of 30% hydrogen peroxide was added and mixed thoroughly.

To detect the POD activity, 1 mL of the enzyme extraction was mixed with 3 mL of the substrate solution, and then the absorbance was measured at 470 nm using a UV–Visible Spectrophotometer.

### 2.5 Detection of fatty acid composition

The fatty acid composition was determined using a Gas Chromatography–Mass Spectrometry (GC–MS, 7890A-5975, Agilent Technologies Inc., Santa Clara, CA, USA). For sample preparation, 1 g of powdered sample was mixed with 1 mL of n-hexane, and 0.25 mL of 2 mol/L KOH-methanol solution. After vertexing for 30 s, the mixture was subjected to ultrasonic extraction at 30°C for 40 min. Following this, 0.25 mL of 2 mol/L hydrochloric acid was added, and vortexed for 1 min. The mixture was then centrifuged at 6000 rpm for 15 min, and the supernatant was collected as sample solution.

GC column separation was conducted using an Agilent HP-5MS UI (30 m × 0.25 mm × 0.25 μm) column. The initial temperature of the oven was set at 130°C, kept for 3 min. Then, it was raised at a rate of 5°C/min to 180°C, kept for 8 min. Subsequently, it was further increased at a rate of 5°C/min to 240°C, kept for 12 min. The carrier gas was helium with a flow rate of 1.0 mL/min, and a split ratio of 10:1. The injection port temperature was maintained at 260°C, while the temperature of the MSD transfer line was set at 280°C. The solvent delay was 1.6 min.

The MS data acquisition was performed using an electron impact ionization source, with ion source temperature of 230°C and quadrupole temperature of 150°C. Full scan analyses were conducted in a range of m/z 45–400.

### 2.6 Evaluation of texture and sensory qualities of cooked rice

The rice grains samples were processed using an automatic rice mill (TM05C, Riichi, Japan). The milled rice was then cooked in a steam cooker (CFXB40B2T-65, Hangzhou, China). The texture of cooked rice was analyzed using a hardness-stickiness texture analyzer (RHS1A, Satake Co. Ltd., Japan). Sensory attributes of the cooked rice were determined using a Cooked Rice Taste Analyzer (STA1B, Satake Co. Ltd., Japan).

### 2.7 Data analysis

The data were expressed as mean ± standard deviation (SD). Statistical analysis was performed using Duncan’s multiple range test to determine significant differences between means. All statistical analyses were conducted using the SPSS software package (SPSS, IL, USA).

## 3 Results and discussion

### 3.1 Indicators relating to lipid metabolism

#### 3.1.1 Fatty acid value

**Figure 1:**
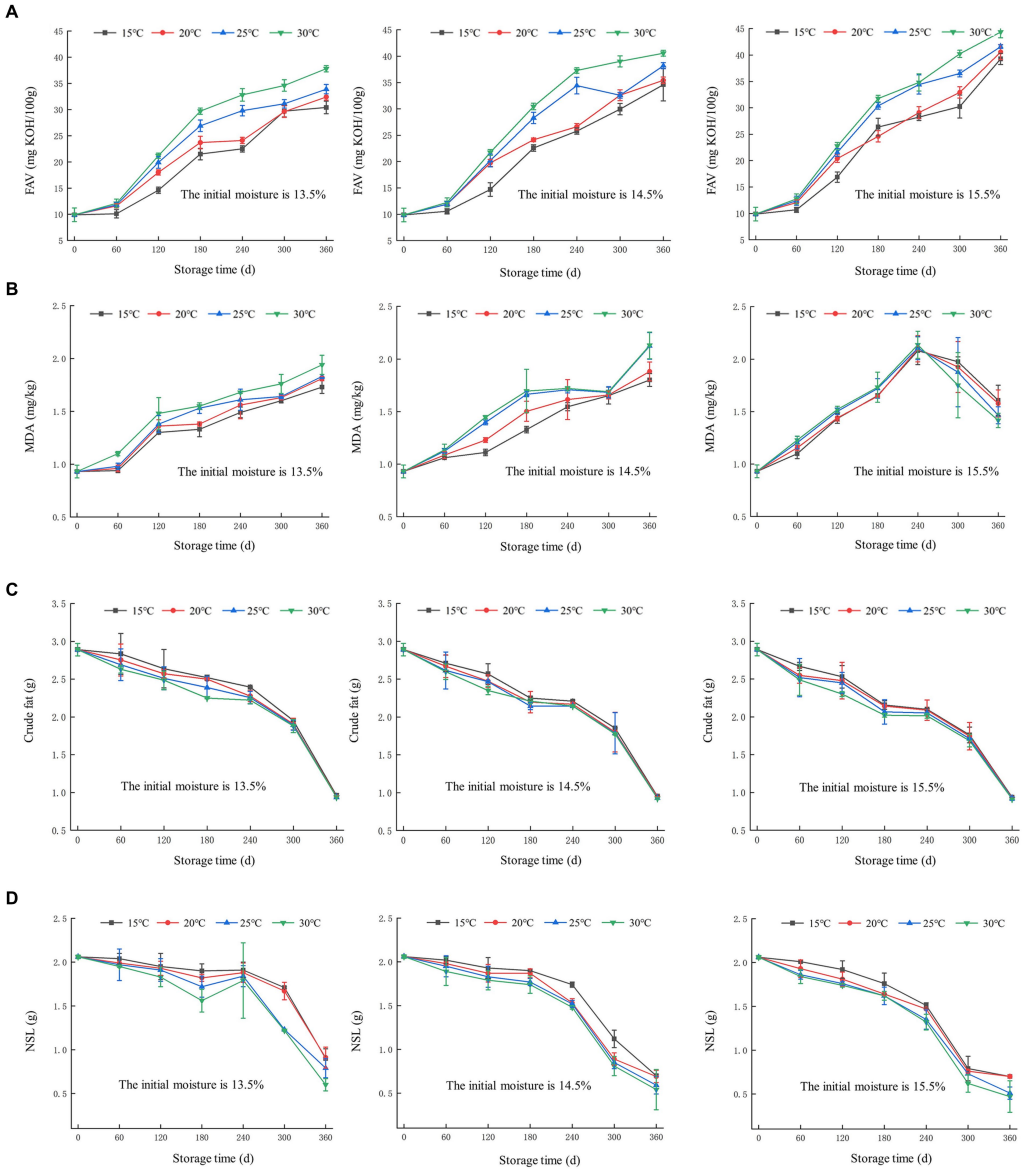
Changes in the indicators relating to lipid metabolism during rice storage. **(A)** Fatty acid value (FAV); **(B)** malondialdehyde (MDA); **(C)** crude fat; **(D)** non-starch lipids (NSL).

FAV is a key indicator for determining the storage quality of rice grains (22). In Figure 1A, there was a noticeable increase in FAV in rice grains throughout the entire storage process, for all three batches with different initial moisture contents. Kim et al. (23) reported that high storage temperature led to a more rapid degradation rates and oxidative reactions of lipids in Japonica rice. FAV exhibited a gradual increase trend within 60 days. However, FAV showed a rapid increase when the storage time ranges from 60 to 180 days. FAV increased faster with the storage temperature rising from 15°C to 30°C. After 240 days of storage, the FAV for batches with initial moisture content of 13.5, 14.5, and 15.5% fell within the ranges of 22.5 ~ 32.8 mg KOH/100 g, 25.8 ~ 37.3 mg KOH/100 g, and 28.2 ~ 34.8 mg KOH/100 g, respectively.

In general, when the FAV of rice exceeds 30 mg KOH/100 g, it is considered unsuitable for storage. After being stored for 300 days, rice sample with initial moisture content of 13.5% reached an unsuitable storage threshold at 30°C. However, rice samples with initial moisture contents of 14.5 and 15.5% reached an unsuitable storage state at relative lower temperature of 25°C. After a storage period of 360 days, the rice sample with the highest initial moisture content of 15.5% have reached a severely unsuitable storage state (37 mg KOH/100 g), indicating that the sample with high initial moisture content was more vulnerable to deterioration in terms of FAV. This can be attributed to the accelerated metabolism rate of lipid substances in rice samples, which leads to an upward trend in FAV.

#### 3.1.2 Malondialdehyde content

The content of MDA is related to the level of free fatty acids. As the degree of lipid peroxidation in rice sample increases, the content of MDA also increases (24, 25). Therefore, the content of MDA can be used as a measure of the degree of lipid peroxidation in rice grains. The MDA analysis results are shown in Figure 1B. With the prolonging of storage time and the increase in storage temperature, the MDA content in rice grain samples showed an upward trend. This result suggested that the degree of lipid peroxidation of rice gradually increased (26). For storage periods of less than 60 days, rice grains samples with initial moisture content of 13.5% showed a slight elevation in MDA levels, while rice samples with initial moisture content of 14.5 and 15.5% exhibited a substantial increase in MDA levels. After 60 days, the MDA levels in all three batches of samples significantly increased with prolonged storage duration. However, when the storage period exceeded 240 days, the MDA content of the batch with the initial moisture content of 15.5% gradually decreased. This could be attributed to the decreased activity of enzyme in cells of rice samples during the later stages of storage, resulting in decreased oxidizing activity of free fatty acids (27). Additionally, high temperatures during the storage may increase the volatility of MDA, leading to a decrease in its content.

#### 3.1.3 Crude fat content

As shown in Figure 1C, the crude fat content in rice samples showed a decreasing trend throughout the entire storage period. The overall decline was relatively slow within 240 days, but it began to decrease rapidly beyond 240 days. Following 360 days of storage, the crude fat content of rice with initial moisture content of 13.5, 14.5, and 15.5% decreased to the range of 1.14 ~ 1.37 g, 0.93 ~ 0.95 g, and 0.91 ~ 0.94 g, respectively, indicating that the initial moisture content has an impact on the crude fat content. In addition, it could be observed that an increase in storage temperature leaded to an accelerated decrease in crude fat content. These results indicated that the higher the temperature and moisture content, the more pronounced the decline in crude fat content, which could be related to the activity of lipase and lipid oxidase.

#### 3.1.4 Non-starch lipid content

NSL refer to the fats on the surface of starch granules. The lipid degradation of rice grains during storage is mainly derived from NSL (28). As shown in Figure 1D, the content of NSL during the storage showed a downward trend. Comparing to the rice grains with higher initial moisture content, samples with lower initial moisture content exhibited a slower rate of decrease. In addition, it was found that there was a significant difference in the variation of NSL content between storage conditions at 15°C and 30°C, indicating that higher temperature promoted the decrease of NSL content. After 360 days of storage, the NSL content of the rice grains with initial moisture contents of 13.5, 14.5, and 15.5% decreased to 0.6 ~ 0.9 g, 0.54 ~ 0.7 g, and 0.47 ~ 0.7 g, respectively.

### 3.2 Enzyme activity

#### 3.2.1 Lipoxygenase activity

**Figure 2:**
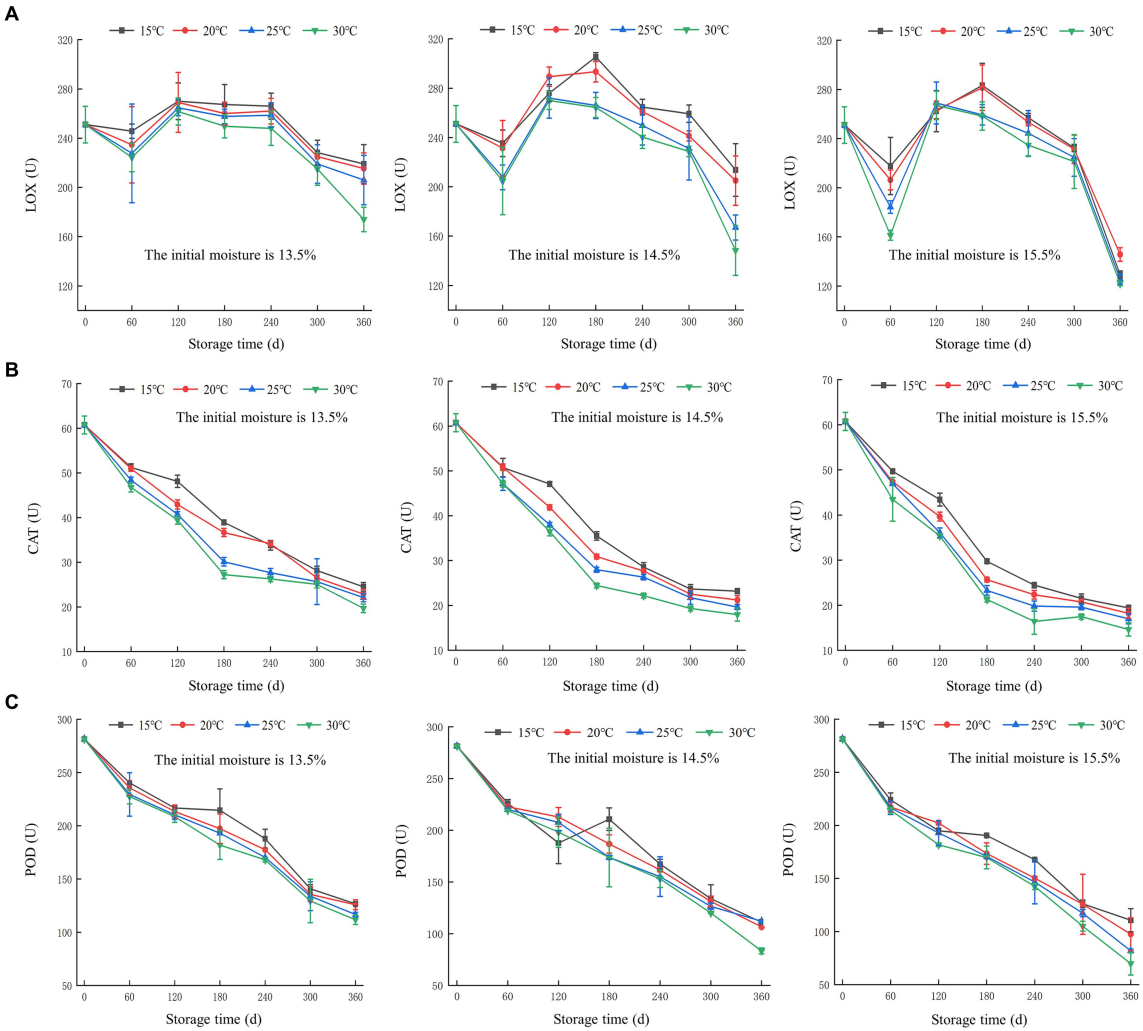
Changes in enzyme activity during rice storage. **(A)** lipoxygenase (LOX); **(B)** catalase (CAT); **(C)** peroxidase (POD).

As shown in Figure 2A, the LOX activity of all three batches showed a trend of initially decreasing, then increasing, and subsequently decreasing again. The decrease in LOX activity within the range of 0 to 60 days was attributed to the involvement of LOX in lipid metabolism, as well as the increase in water-soluble acids in the rice grains, leading to enzyme inhibition (29). However, the enzyme protein did not undergo complete denaturation and deactivation. During the period of 60 to 180 days, the LOX in the reversible inhibited state returned to an activated state, leading to a subsequent increase in activity. After 180 days of storage, due to the accumulation of peroxides and water-soluble acids, the LOX completely deactivated, resulting in the recurrence of a downward trend.

At 30°C, the LOX activity of rice grains with an initial moisture content of 13.5% decreased from 251 U to 174 U, whereas the LOX activity of 15.5% batch decreased from 251 U to 122 U. This proved that the LOX activity decreased at a faster rate in rice grains with higher initial moisture content. In addition, it could be concluded that the LOX activity decreased more rapidly in rice grains stored under high temperature conditions.

#### 3.2.2 Catalase activity

As shown in Figure 2B, during the storage, the CAT activity gradually decreased. In addition, a faster rate of decrease could be observed under high temperature conditions. In the beginning, CAT activity was measured at 60.75 U. Following 360 days of storage, the CAT activity for the batches with initial moisture content of 13.5, 14.5, and 15.5%, reached the range of 27.23 ~ 38.93 U, 24.45 ~ 35.5 U, and 21.28 ~ 29.75 U, respectively. Comparing to the group with a high moisture content, lower initial moisture content showed a slower decrease in CAT activity, proving that the advantages of reducing moisture content for preserving rice quality.

#### 3.2.3 Peroxidase activity

As shown in Figure 2C, the overall trend exhibited a decrease in POD activity in rice grains at different storage temperatures for all three groups. At 30°C, the POD activity decreased by 169.73 U, 198 U, and 211.6 U for the rice grains samples with the initial moisture content of 13.5, 14.5, and 15.5%, respectively. This indicated that higher moisture content leaded to a more rapid decrease in POD activity. Furthermore, at the same initial moisture level, the sample stored at 30°C exhibited a greater decrease in POD activity of 5 ~ 30 U compared to those stored at 15°C, indicating that high temperature can accelerate the decline in enzyme activity.

### 3.3 Fatty acid composition

In order to evaluate the changes in fatty acids during the storage process of “Xiadao No.1” rice grains sample, five types of typical fatty acids, including lauric acid, palmitic acid, stearic acid, oleic acid, and linoleic acid, were analyzed. As shown in Table 1, linoleic acid, oleic acid, and palmitic acid are the major fatty acid components in rice sample. This showed similar results with those reported by Nakamura et al. (30). After storage, the content of linoleic acid showed a downward trend, while the other four types of fatty acids increased. This could be explained by the fact that LOX primarily acts on linoleic acid as a substrate, resulting in the gradual decrease in linoleic acid (31). Yoon et al. reported that the palmitic and oleic acid contents of rice samples increased after storage, and the linoleic acid content showed a considerable decrease (32). No significant correlations were observed between the content of fatty acids and either the storage temperature or initial moisture content.

**Table 1:** Changes in fatty acid composition during rice storage.

Moisture content (%)	Types of fatty acids	Storage temperature (°C)	Storage time (d)					
			0	60	120	180	240	300
13.5	Myristic acid	15	0.70	0.84	1.03	1.14	1.25	1.24
		20		0.93	1.18	1.15	1.28	1.34
		25		0.93	1.20	1.21	1.36	1.34
		30		1.04	1.30	1.26	1.39	1.54
	Stearic acid	15	1.18	1.69	1.62	1.74	2.06	2.16
		20		1.20	1.25	1.61	1.79	1.76
		25		1.18	1.30	1.62	1.83	1.75
		30		1.29	1.43	1.64	1.86	1.96
	Palmitic acid	15	18.68	23.14	23.05	25.61	26.26	26.41
		20		25.51	26.56	26.28	26.98	27.20
		25		25.63	27.50	26.72	28.12	30.56
		30		23.89	27.78	28.92	28.58	28.66
	Oleic acid	15	32.60	34.61	36.73	35.54	37.41	38.62
		20		33.33	35.02	36.90	37.87	37.42
		25		34.09	34.73	35.55	35.66	35.00
		30		33.14	33.52	33.66	34.91	33.85
	Linoleic acid	15	46.85	36.69	37.57	35.97	33.03	31.57
		20		39.03	36.00	34.06	32.07	32.28
		25		38.17	35.27	34.90	33.03	31.35
		30		40.63	35.97	34.52	33.26	34.00
14.5	Myristic acid	15	0.70	0.93	1.05	1.17	1.17	1.27
		20		0.82	1.01	1.04	1.15	1.37
		25		0.87	1.04	1.01	1.27	1.36
		30		0.85	1.08	0.92	1.28	1.43
	Stearic acid	15	1.18	1.60	1.55	1.93	2.04	1.97
		20		1.48	1.94	1.63	2.08	2.04
		25		1.54	1.59	1.82	1.84	2.06
		30		1.56	1.74	1.77	2.02	2.17
	Palmitic acid	15	18.68	22.64	24.17	25.02	24.22	25.75
		20		23.07	23.97	23.95	23.85	25.10
		25		22.95	23.42	23.20	26.44	26.19
		30		23.07	26.33	25.87	27.40	27.34
	Oleic acid	15	32.60	36.30	36.17	37.03	38.68	39.62
		20		35.35	37.55	37.73	36.70	40.09
		25		36.00	37.60	37.21	39.17	39.33
		30		34.98	36.65	35.89	37.80	39.04
	Linoleic acid	15	46.85	38.54	37.06	34.86	33.88	31.39
		20		39.29	35.52	35.65	36.21	31.40
		25		38.64	36.35	36.76	31.28	31.07
		30		39.54	34.20	32.17	31.50	30.01
15.5	Myristic acid	15	0.70	1.01	1.23	1.02	0.97	1.26
		20		0.80	0.93	0.80	0.95	1.18
		25		0.78	0.98	1.08	1.09	1.22
		30		0.84	1.01	1.06	1.05	1.27
	Stearic acid	15	1.18	1.47	1.54	1.79	1.96	1.91
		20		1.58	1.64	1.71	1.94	1.93
		25		1.66	1.89	2.12	2.17	2.19
		30		1.71	1.86	2.02	2.22	2.29
	Palmitic acid	15	18.68	21.70	21.56	24.83	24.17	24.72
		20		21.13	23.92	23.86	24.93	24.77
		25		21.24	23.86	23.50	24.90	25.28
		30		23.10	25.44	25.63	25.52	26.21
	Oleic acid	15	32.60	33.44	36.59	35.70	36.94	37.49
		20		33.95	36.04	38.15	37.43	38.68
		25		34.82	35.99	34.10	36.94	37.12
		30		34.35	36.71	35.63	36.41	38.23
	Linoleic acid	15	46.85	42.38	39.08	36.66	35.95	34.63
		20		42.54	37.47	35.50	34.75	33.45
		25		41.50	37.28	39.20	34.89	34.20
		30		39.99	34.97	35.65	34.80	31.99

### 3.4 Texture and sensory attributes of cooked rice

After 360 days of storage, the rice grains were prepared as cooked rice, and the texture and sensory qualities were evaluated. Hardness is an important indicator of rice aging (33). After storage, the hardness of rice increased. This could be attributed to the interaction between the free fatty acids generated during the storage process and amylose in rice, which decreased the gelatinization ability of amylose during cooking (14). As a result, the strength of starch granules increased, leading to the increased hardness of cooked rice. The lowest hardness of cooked rice was observed for rice sample stored under low temperature conditions. In general, the taste value was highest for the samples stored under 15°C, and lowest for those stored under 30°C. The result of sensory scores (comprehensive score) were generally consistent with the hardness and taste value. These results demonstrated that lower storage temperature and initial moisture content of the rice grains was beneficial for the storage of rice (see Table 2).

**Table 2:** Changes of taste quality after 360 days of storage.^*^

Storage condition		Hardness	Taste value	Sensory score
Moisture content (%)	Temperature(°C)			
Fresh sample	–	0.34 ± 0.02^a^	6.70 ± 0.14^a^	72.15 ± 1.34^a^
13.5	15	0.57 ± 0.02^b^	5.05 ± 0.07^b^	60.00 ± 1.41^b^
	20	0.58 ± 0.06^b^	4.60 ± 0.85^b^	55.95 ± 5.44^b^
	25	0.64 ± 0.12^b^	2.35 ± 0.35^c^	39.55 ± 0.64^c^
	30	0.68 ± 0.10^b^	2.05 ± 0.78^c^	38.05 ± 6.15^c^
14.5	15	0.62 ± 0.05^a^	5.00 ± 0.00^b^	60.30 ± 5.23^b^
	20	0.73 ± 0.06^a^	4.20 ± 0.14^c^	46.60 ± 5.80^b^
	25	0.73 ± 0.05^a^	2.50 ± 0.28^d^	43.45 ± 6.15^bc^
	30	0.66 ± 0.34^a^	2.00 ± 0.28^d^	34.25 ± 1.06^c^
15.5	15	0.60 ± 0.13^ab^	4.05 ± 0.21^b^	51.60 ± 2.69^b^
	20	0.64 ± 0.11^ab^	3.40 ± 0.99^b^	51.55 ± 0.21^b^
	25	0.73 ± 0.19^b^	2.85 ± 0.49^bc^	40.80 ± 1.98^c^
	30	0.80 ± 0.05^b^	1.75 ± 0.35^c^	36.95 ± 2.05^c^

^*^Data were expressed as means ± SD. Means within a column that had the same letter were not significantly different (α = 0.05).

## 4 Conclusion

During the storage period, the high-quality rice gradually aged, which is manifested in the increase of FAV, and cooked rice hardness and the decrease of total fat and NSL content, POD and CAT activities, and sensory score of cooked rice. The elevation of storage temperature and moisture content accelerated the aging of high-quality rice. After 360 days of storage, sensory attributes of cooked rice dramatically declined. Most rice samples showed a comprehensive score of 35 ~ 60 except the rice with moisture contents of 13.5 and 14.5% stored at 15°C. These results suggested that storage condition of low temperature (15°C) and low initial moisture content (13.5%) can retard the aging process of rice. These results can provide theoretical and technical reference for the better storage of high-quality rice.

## Data availability statement

The raw data supporting the conclusions of this article will be made available by the authors, without undue reservation.

## Author contributions

DZ: Investigation, Writing – original draft. TW: Investigation, Writing – original draft. XL: Writing – review & editing. JB: Writing – original draft. WZ: Funding acquisition, Validation, Writing – review & editing. XZ: Validation, Writing – review & editing. PW: Conceptualization, Methodology, Writing – original draft. ZS: Conceptualization, Methodology, Writing – review & editing.
